# Synthesis, Antimycobacterial, Antifungal and Photosynthesis-Inhibiting Activity of Chlorinated *N*-phenylpyrazine-2-carboxamides †

**DOI:** 10.3390/molecules15128567

**Published:** 2010-11-26

**Authors:** Martin Dolezal, Jan Zitko, Zdenek Osicka, Jiri Kunes, Marcela Vejsova, Vladimir Buchta, Jiri Dohnal, Josef Jampilek, Katarina Kralova

**Affiliations:** 1 Faculty of Pharmacy in Hradec Kralove, Charles University in Prague, Heyrovskeho 1203, Hradec Kralove, 500 05, Czech Republic; E-Mails: jan.zitko@faf.cuni.cz (J.Z.); jiri.kunes@faf.cuni.cz (J.K.); marcela.vejsova@faf.cuni.cz (M.V.); 2 Bioveta a.s., Komenskeho 212, 683 23 Ivanovice na Hane, Czech Republic; E-Mail: osicka.zdenek@bioveta.cz (Z.O.); 3 Department of Clinical Microbiology, Faculty of Medicine and University Hospital, Charles University in Prague, Sokolska 581, Hradec Kralove, 500 05, Czech Republic; E-Mail: buchta@fnhk.cz (V.B.); 4 Zentiva k.s., U Kabelovny 130, 102 37 Prague 10, Czech Republic; E-Mails: jiri.dohnal@zentiva.cz (J.D.); josef.jampilek@zentiva.cz (J.J.); 5 Faculty of Pharmacy, University of Veterinary and Pharmaceutical Sciences, Palackeho 1/3, 61242 Brno, Czech Republic; 6 Institute of Chemistry, Faculty of Natural Sciences, Comenius University, Mlynska Dolina CH-2, 842 15 Bratislava, Slovak Republic; E-Mail: kralova@fns.uniba.sk (K.K.)

**Keywords:** pyrazinecarboxamides, lipophilicity, *in vitro* antimycobacterial activity, *in vitro* antifungal activity, spinach chloroplasts, PET inhibition, structure–activity relationships

## Abstract

A series of sixteen pyrazinamide analogues with the -CONH- linker connecting the pyrazine and benzene rings was synthesized by the condensation of chlorides of substituted pyrazinecarboxylic acids with ring-substituted (chlorine) anilines. The prepared compounds were characterized and evaluated for their antimycobacterial and antifungal activity, and for their ability to inhibit photosynthetic electron transport (PET). 6-Chloro-*N*-(4-chlorophenyl)pyrazine-2-carboxamide manifested the highest activity against *Mycobacterium tuberculosis* strain H37Rv (65% inhibition at 6.25 μg/mL). The highest antifungal effect against *Trichophyton mentagrophytes*, the most susceptible fungal strain tested, was found for 6-chloro-5-*tert*-butyl-*N*-(3,4-dichlorophenyl)pyrazine-2-carboxamide (MIC = 62.5 μmol/L). 6-Chloro-5-*tert*-butyl-*N*-(4-chlorophenyl)pyrazine-2-carboxamide showed the highest PET inhibition in spinach chloroplasts (*Spinacia oleracea* L.) chloroplasts (IC_50_ = 43.0 μmol/L). For all the compounds, the relationships between the lipophilicity and the chemical structure of the studied compounds as well as their structure–activity relationships are discussed.

## 1. Introduction

**Figure 1:**
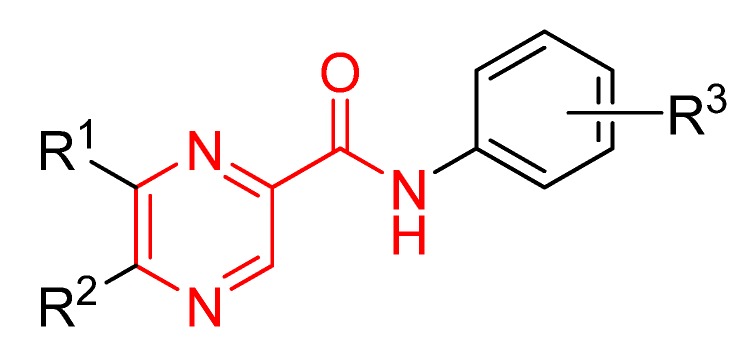
Pyrazinamide (red colour) structure modification (black colour).

Compounds possessing a -CONH- moiety simulating a peptide bond in their molecule show a broad range of biological effects. Pyrazinamide, with its simple structure, provides a good opportunity for further modification with a view to increasing its antimycobacterial activity. We have prepared and studied several series of the pyrazinamide analogues with the -CONH- linker connecting the pyrazine and benzene rings. All compounds were assayed *in vitro* against major *Mycobacterium* and various fungal species [1,2,3,4,5,6]. Some compounds were found to exhibit photosynthesis-inhibiting activity [2,5,7,8]. Various *N*-substituted amides of pyrazinecarboxylic acid were prepared and evaluated as potential abiotic elicitors [9,10,11,12]. Introducing of halogens (-Cl, -F, -CF_3_) was the most successful structural modification. *N*-(3-Trifluoromethylphenyl)pyrazine-2-carboxamide, 5-*tert*-butyl-6-chloro-*N*-(3-trifluoromethylphenyl)pyrazine-2-carboxamide, and *N*-(3-iodo-4-methylphenyl)pyrazine-2-carbox-amide have shown the highest activity against *M. tuberculosis* H37Rv (MIC = 3.13-6.25 μg/mL) [5]. This paper describes the preparation, biological evaluation and structure–activity relationship studies of a series of chlorinated pyrazinamide analogues. We synthesized in preference compounds with lipophilic and/or electron-withdrawing substituents on the benzene moiety (R^3^, chlorine), and the compounds with the substitution on the pyrazine nucleus with R^1^ (hydrogen, chlorine) and/or R^2^ (hydrogen, *tert*-butyl) moiety (see Figure 1).

Many low molecular weight drugs cross biological membranes through passive transport, which strongly depends on their lipophilicity, which is one of the most important physical properties of biologically active compounds. It influences the transport of a molecule through cellular membranes, because drugs cross biological barriers most frequently through passive transport, which strongly depends on their lipophilicity. Lipophilicity is a property that has a major effect on absorption, distribution, metabolism, excretion, and toxicity (ADME/Tox) properties as well as pharmacological activity. Lipophilicity has been studied and applied as an important drug property for decades [13].

The lipophilicity of pyrazinamide is quite low (log *P* = -1.31/CLog*P* = -0.67632), therefore in an effort to increase it we have chosen hydrophobic electron-withdrawing (chlorine), and bulky substitutents on the pyrazine (*tert*-butyl), and the combination of substituents (chlorine) on the benzene part. Distributive π parameters are firmly established as the parameter of choice for correlating both binding to biological macromolecules and transport through a biological system. The determined π parameters of substituents can be used for describing relationships between physico-chemical properties and biological activity of prepared ring-substituted pyrazine-based compounds [14,15]. The distributive π parameters of individual substituents are listed for the mentioned studied compounds. Although all the discussed compounds are relatively simple structures substituted within the series only by chlorine, interesting intramolecular interactions influencing lipophilicity were observed, probably due to the simultaneous presence of a pyrazine ring and a carboxamide moiety.

The aim of this work was to examine the structure–activity relationships (SAR) in the mentioned series, *i.e.* to continue in the study of the substituent variability influence on the biological activity, and to determine the importance of increased lipophilic properties for biological effect of the newly prepared substituted pyrazinecarboxamides.

## 2. Results and Discussion

### 2.1. Chemistry

**Scheme 1:**
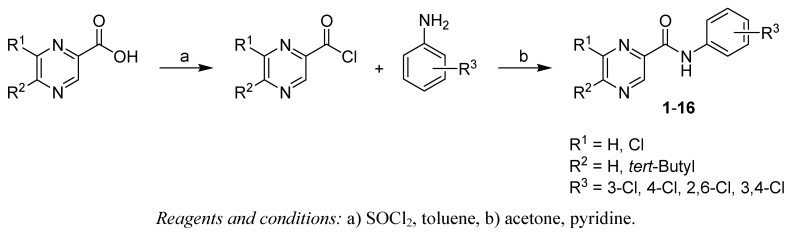
Synthetic pathway and general formula of prepared amides **1-16**.

Condensation of the chlorides of pyrazine-2-carboxylic, 6-chloropyrazine-2-carboxylic, 5-*tert*-butyl-pyrazine-2-carboxylic or 5-*tert*-butyl-6-chloropyrazine-2-carboxylic acids with commercially available ring-substituted anilines yielded a series of 16 substituted amides **1**-**16** [2,3,5]. All studied compounds were prepared according to Scheme 1.

### 2.2. Lipophilicity

**Figure 2:**
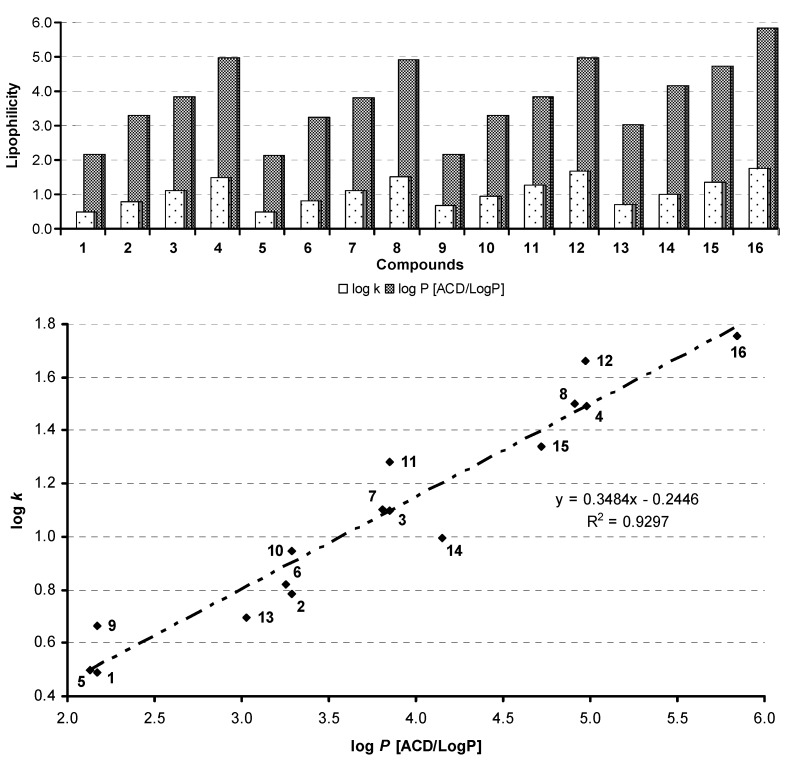
Match of the calculated log *P* data with the experimentally found log *k* values.

Lipophilicity parameters (log *P*) of the compounds **1**-**16** were calculated using the commercially available program ACD/LogP and also measured by means of the RP-HPLC determination of capacity factors *k* with subsequent calculation of log *k*. The procedure was performed under isocratic conditions with methanol as an organic modifier in the mobile phase using an end-capped non-polar C_18_ stationary RP column. The results are shown in Table 1 and illustrated in Figure 2.

**Table 1:** Comparison of the calculated lipophilicity (log *P*) with the determined log *k* values of the discussed pyrazinecarboxamides **1**-**16**, as well as the determined distributive parameters π calculated from log *k*. 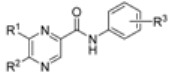

**Comp.**	R^1^	R^2^	R^3^	log *k*	log *P* ACD/LogP	π_determined_ Pyr/Ph	σ [15,16]
**1**	H	H	3-Cl	0.4914	2.17 ± 0.41	0.00/0.08	0.373
**2**	Cl	H	3-Cl	0.7864	3.29 ± 0.42	0.30/0.10	0.373
**3**	H	(CH_3_)_3_C	3-Cl	1.0996	3.85 ± 0.41	0.61/0.26	0.373
**4**	Cl	(CH_3_)_3_C	3-Cl	1.4896	4.98 ± 0.43	1.00/0.25	0.373
**5**	H	H	4-Cl	0.4987	2.13 ± 0.41	0.00/0.09	0.227
**6**	Cl	H	4-Cl	0.8185	3.25 ± 0.42	0.32/0.13	0.227
**7**	H	(CH_3_)_3_C	4-Cl	1.1043	3.81 ± 0.41	0.61/0.16	0.227
**8**	Cl	(CH_3_)_3_C	4-Cl	1.5015	4.91 ± 0.43	1.00/0.26	0.227
**9**	H	H	2,6-Cl	0.6656	2.17 ± 0.41	0.00/0.25	0.40
**10**	Cl	H	2,6-Cl	0.9456	3.29 ± 0.43	0.30/0.28	0.40
**11**	H	(CH_3_)_3_C	2,6-Cl	1.2802	3.85 ± 0.42	0.61/0.34	0.40
**12**	Cl	(CH_3_)_3_C	2,6-Cl	1.6631	4.97 ± 0.44	1.00/0.42	0.40
**13**	H	H	3,4-Cl	0.6962	3.03 ± 0.42	0.00/0.30	0.60
**14**	Cl	H	3,4-Cl	0.9950	4.15 ± 0.44	0.28/0.31	0.60
**15**	H	(CH_3_)_3_C	3,4-Cl	1.3395	4.72 ± 0.43	0.62/0.40	0.60
**16**	Cl	(CH_3_)_3_C	3,4-Cl	1.7563	5.84 ± 0.45	1.04/0.51	0.60

Compounds **1**, **5**, **9** show the lowest lipophilicity, whereas compound **16** possesses the highest lipophilicity. The calculated log *P* data and the determined log *k* parameters correspond to the expected lipophilicity increasing within individual series of compounds (pyrazine < 6-chloropyrazine < 5-*tert*-butylpyrazine < 6-chloro-5-*tert*-butylpyrazine derivatives). This dependence is approximately linear.

Some significant differences between the experimental values log *k* and the calculated parameters log *P* at compounds **9**-**12** with substitution in *ortho*-position (2,6-Cl) were observed. Better correlation at derivatives with chloro substitution in position 3 or 4 was found. Lipophilicity increases according to substitution in anilide part of the molecule this way: 3-Cl < 4-Cl < 2,6-Cl < 3,4-Cl. It can be assumed that log *k* values specify lipophilicity within the individual series of the studied compounds more precisely than calculated log *P* data at compounds with the *ortho* substitution in the benzene part.

The distributive parameter π describes the lipophilicity contribution of individual moieties substituted on some skeleton. The distributive constants π of individual substituents are dependent on the basic skeleton (aliphatic, aromatic, heteroaromatic), as well as on the character of the heteroaromatic system. A number of distributive parameters π for various substituents for all three substituent positions in the benzene ring has been described [14,15]. The determined π parameters of substituents can be used for describing relationships between the physico-chemical properties and activity of prepared compounds. Due to similarity of the determined π phenyl parameters for compounds **1**-**4** (3-Cl) and **5**-**8** (4-Cl) it can be predicted that these individual/independent positions/substitutions do not show any intramolecular interactions between chlorine and the pyrazine core or carboxamide moiety contrary to disubstituted compounds **13**-**16** (3,4-Cl), where both chlorine atoms interact with each other. Results from Table 1 show quite different behaviours of both chlorine atoms in **9**-**12** (2,6-Cl).

### 2.3. In vitro antimycobacterial evaluation

All compounds were assayed *in vitro* against *M. tuberculosis* H37Rv. In the tuberculosis antimicrobial acquisition and coordinating facility (TAACF) program [17] the compounds showing >90% inhibition in this preliminary screen (*i.e.* MIC < 6.25 µg/mL) are further evaluated to determine their actual minimum inhibitory concentration (MIC) in the MABA. None of the tested derivatives overcame this limit, see Table 2.

**Table 2:** The antimycobacterial activity (%) of the compounds in comparison with the standard pyrazinamide (PZA), the *in vitro* antifungal (IC_50_) activity of the compounds against *Trichophyton mentagrophytes* (determined after 72h/120h) compared with the fluconazole (FLU) standard and IC_50_ values of compounds **1**-**16** related to photosynthetic electron transport (PET) inhibition in spinach chloroplasts in comparison with the 3-(3,4-dichlorophenyl)-1,1-dimethylurea (DCMU) standard. (MIC = minimum inhibitory concentration, ND = not determined due to their low solubility in the testing medium).

**Comp.**	Antimycobacterial evaluation Inhibition [%]	Antifungal susceptibility MIC [µmol/L] *^a^*	PET inhibitionIC_50_ [μmol/L]
**1**	14	500/>500	290.1
**2**	14	125/125	262.0
**3**	0	>250/>250	95.5
**4**	0	>250/>250	100.7
**5**	4	>250/>250	1,523
**6**	**65**	>500/>500	486.0
**7**	0	>250/>250	ND
**8**	24	>250/>250	**43.0**
**9**	0	>500/>500	ND
**10**	0	>250/>250	829.3
**11**	0	250/250	153.0
**12**	0	125/125	61.0
**13**	8	>250/>250	ND
**14**	**61**	125/250	104.8
**15**	15	125/125	ND
**16**	0	**62.5**/**62.5**	130.1
**PZA**	*^b^*	–	–
**FLU**	–	1.95/3.91	–
**DCMU**	–	–	1.9

*^a^* The MIC determination for moulds-fungi is determined as IC_50_ value; *^b^* MIC = 12.5 µg/mL [18].

Only 6-chloro-*N*-(4-chlorophenyl)pyrazine-2-carboxamide (**6**) possessed activity against *M. tuberculosis* strain H37Rv 65% inhibition at 6.25 μg/mL and 6-chloro-*N*-(3,4-dichlorophenyl)pyrazine-2-carboxamide (**14**) showed 61%. Although their activity did not warrant progression to phase II screening, medium-active compounds such as **6**, **14** should not be ignored, because their chemical analogues and alterations in physico-chemical properties may confer some positive changes in biological effects.

With respect to the mostly low-active compounds, only some general structure–activity relationship (SAR) aspects within this series of these specific substituted compounds can be proposed. There is considered the positive effect of chlorine atom for both pyrazine (C_(6)_ position) and benzene ring (especially in C_(4)_ position), and the negative influence of alkyl introduction to the pyrazine nucleus. Lipophilicity of the compounds has an important role. The above discussed compounds **6** and **14** comply with both π_Pyr_ and π_Ph_ limits for antimycobacterial activity within this series of the chlorine substituted *N*-phenylpyrazine-2-carboxamides **1**-**16**.

### 2.4. In vitro antifungal susceptibility testing

The evaluation of *in vitro* antifungal activity of the synthesized compounds was performed against eight fungal strains, but only moderate activity against *Trichophyton mentagrophytes* is showed in Table 2. Generally, all the compounds afforded only slight antifungal activity caused by the low solubility of compounds in the testing medium and their precipitation during the incubation period, therefore no thorough structure-activity relationships could be established. More lipophilic disubstituted compounds (log *k* < 1) with chlorine atoms especially in positions 3 and 4 on the benzene part of molecule possessed some weak antifungal activity, and 6-chloro-5-*tert*-butyl-*N*-(3,4-dichloro-phenyl)pyrazine-2-carboxamide (**16**) exhibited MIC = 62.5 μmol/L (log *k* = 1.7563) against *T. mentagrophytes*, the most susceptible fungal strain tested within the discussed series of the compound. This activity is only modest in comparison with fluconazole (MIC = 3.91 μmol/L after 120 h, see Table 2).

### 2.5. Inhibition of photosynthetic electron transport (PET) in spinach chloroplasts

Over 50% of commercially available herbicides act by reversibly binding to photosystem II (PS II), a membrane-protein complex in the thylakoid membranes which catalyses the oxidation of water and the reduction of plastoquinone [19] and thereby inhibit photosynthesis [20,21,22]. Some organic compounds, e.g., substituted anilides of 2,6-disubstituted pyridine-4-thiocarboxamides [23] were found to interact with tyrosine radicals Tyr_Z_ and Tyr_D_ which are situated in D_1_ and D_2_ proteins on the donor side of PS II and due to this interaction interruption of the photosynthetic electron transport occurred. On the other hand, 6-chloro-5-*tert-*butyl-*N*-(4-hydroxyphenyl)pyrazine-2carboxamide and 6-chloro-5-*tert*-butyl-*N*-(5-chloro-3-hydroxyphenyl)pyrazine-2carboxamide interacted only with the D^+^ intermediate [8].

All the discussed compounds were tested for their photosynthetic electron transport (PET) inhibition in spinach chloroplasts and they showed some wide-range activity, see Table 2. The IC_50_ values related to PET inhibition could not be determined for **7**, **9**, **13** and **15** due to precipitation of the compounds during the experiments. The activity of the majority of the studied compounds was moderate or low relative to the standard.

The most effective inhibitor from the series was 6-chloro-5-*tert*-butyl-*N*-(4-chlorophenyl)pyrazine-2-carboxamide (**8**, IC_50_ = 43 μmol/L), as measured on photosynthetic electron transport (PET) in spinach (*Spinacia oleracea* L.) chloroplasts, see Table 2. It can be again considered the positive effect of chlorine atom for both pyrazine (C_(6)_ position) and benzene ring (especially in C_(4)_ position), as discussed above. Influence of *tert*-butyl moiety introduction on pyrazine demonstrated the positive effect, contrary to negative influence on antimycobacterial activity. It is evident from Table 1 and Table 2 that the lipophilicity of the compound is determining for PET inhibition. It seems to be fundamental that PET inhibition is conditioned by high π_Pyr_ parameter (Table 1), which is around 1 (substituted both by chlorine in the C_(6)_ and by *tert*-butyl in the C_(5)_ positions of pyrazine). Substitution of pyrazine core completes an advantageous substitution of benzene ring, especially in the C_(3)_ or C_(4)_ positions. Disubstitution of both C_(3)_, C_(4)_ positions on benzene increases lipophilicity and at the same time depresses water-solubility. The sum of both π_Pyr_, π_Ph_ of substituents present in each compound can be *de facto* considered log *k* value. When values of PET inhibition with log *k* are compared, it can be stated that an increase in lipophilicity to log *k* ~ 1.50 enhances the effectiveness of PET-inhibiting activity, but subsequent increasing lipophilicity of the compounds decreases their activity.

Beside lipophilicity parameters, the contribution of electronic properties of phenyl substituents to PET-inhibiting activity was investigated as well. These properties, expressed as Hammett's σ constants are described in Table 1 [15,16].

Despite the relatively low inhibitory activity of the studied compounds, the correlations between log (1/IC_50_) and lipophilicity characteristics (log *k*, log *P*, π_Pyr_, π_Ph,_ (π_pyr+_π_Ph_) or Hammett's constants (σ) of the R^3^ substituent were calculated. The importance of compound lipophilicity was for the inhibitory activity (IC_50_ in µmol/L) of compounds much more significant (Eqs. 1 and 3) than the electronic properties of the R^3^ substituent. Introduction of σ parameter in the correlations did not improve the results of statistical analysis (Eqs. 2 and 4) indicating that this parameter is not significant for PET-inhibiting activity:

log (1/IC_50_) = 0.822 (± 0.225) log *k* + 2.808 (± 0.267)

r = 0.756, s = 0.321, F =13.31, n = 12 (1)

log (1/IC_50_) = 0.807 (± 0.254) log *k* + 0.137 (± 0.883) σ + 2.771 (± 0.363)

r = 0.756, s = 0.338, F =6.02, n = 12 (2)

log (1/IC_50_) = 0.314 (± 0.083) log *P* + 2.500 (± 0.334)

r = 0.769, s = 0.314, F =14.45, n = 12 (3)

log (1/IC_50_) = 3.254 (± 0.098) log *P* - 0.236 (± 0.903) σ + 2.544 (± 0.390)

r = 0.771, s = 0.330, F =6.59, n = 12 (4)

Similarly, correlations between PET-inhibiting activity and distributive lipophilicity parameters π_Pyr,_ π_Ph_ as well as their sum [π_pyr+_π_phenyl_] (Eqs. 5-7) were performed:

log (1/IC_50_) = 0.920 (± 0.237) π_pyr_ + 3.225 (± 0.156)

r = 0.775, s = 0.310, F = 15.06, n = 12 (5)

log (1/IC_50_) = 1.954 (± 0.904) π_Ph_ + 3.227 (± 0.257)

r = 0.564, s = 0.405, F = 4.67, n = 12 (6)

log (1/IC_50_) = 0.606 (± 0.223) (π_Pyr+_π_Ph_) + 3.291 (± 0.191)

r = 0.652, s = 0.372, F = 7.39, n = 12 (7)

From the results it is evident that for PET-inhibiting activity predominantly the lipophilicity of substituents on the pyrazine ring (R^1^ and R^2^) is determinant. Lower values of correlation coefficients could be affected by relatively low inhibitory activity of the studied compound as well as with decreased aqueous solubility of more lipophilic compounds.

## 3. Experimental

### 3.1. General

All organic solvents used for the synthesis were of analytical grade. The solvents were dried and freshly distilled under argon atmosphere. The reactions were monitored and the purity of the products was checked by TLC (Merck UV 254 TLC plates, Darmstadt, Germany) using developing solvents hexane/ethyl acetate (9:1). Compounds were purified using a Flash Master Personal Chromatography System (Argonaut Technologies, Redwood City, CA, USA), with hexane/ethyl acetate (9:1) as solvent and Kieselgel 60, 0.040-0.063 mm (Merck, Darmstadt, Germany) as the column sorbent. The melting points were determined using a Melting Point Apparatus SMP 3 (BIBBY Stuart Scientific, UK) and are uncorrected. Elemental analyses were performed on an automatic microanalyser CHNS-O CE instrument (FISONS EA 1110, Milano, Italy). Infrared spectra were recorded on a Nicolet™ Impact 400 FT-IR Spectrometer (Thermo Scientific, USA) in KBr pellets. All ^1^H- and ^13^C-NMR Spectra were recorded on a Varian Mercury – Vx BB 300 (300 MHz for ^1^H and 75 MHz for ^13^C), Varian (Palo Alto CA, USA) in CDCl_3_ solutions at ambient temperature. Chemical shifts are reported in ppm (δ) using internal Si(CH_3_)_4_ as the reference, with diffuse, easily exchangeable signals being omitted.

### 3.2. Synthesis

#### 3.2.1. General procedure for the synthesis of compounds **1-16**

A mixture of acid, *i.e. ,* pyrazinecarboxylic, 6-chloropyrazine-2-carboxylic [2], 5-*tert*-butylpyrazine-2-carboxylic [3] or 5-*tert-*butyl-6-chloropyrazine-2-carboxylic [3] acid, respectively, (50.0 mmol) and thionyl chloride (5.5 mL, 75.0 mmol) in dry toluene (20 mL) was refluxed for about 1 h. Excess of thionyl chloride was removed by repeated evaporation with dry toluene *in vacuo*. The crude acyl chloride dissolved in dry acetone (50 mL) was added dropwise to a stirred solution of the corresponding substituted amine (50.0 mmol) and pyridine (50.0 mmol) in 50 mL of dry acetone keeping at the room temperature. After the addition was complete, stirring continued for the next 30 min. Then the reaction mixture was poured into 100 mL of cold water and the crude amide was collected and purified by the column chromatography. The studied compounds **1**-**16** are presented in the Table 1. The synthesis, physico-chemical data and analytical parameters of several of these compounds were described elsewhere (derivatives **5**-**8** [3] and **13**-**16** [7]).

*Pyrazine-2-carboxylic acid (3-chlorophenyl)amide* (**1**). Yield: 73%; m.p. 139.0-140.0 °C; Anal. Calcd. for C_11_H_8_ClN_3_O (233.7): 56.54% C, 3.45% H, 17.98% N; found: 56.53% C, 3.51% H, 18.03% N; IR (cm^-1^): 3435 (N-H), 1673 (C=O); ^1^H-NMR δ: 9.68 (bs, 1H, NH), 9.50 (s, 1H, H3), 8.83 (d, 1H, *J* = 2.19 Hz, H6), 8.62-8.57 (m, 1H, H5), 7.92-7.86 (m, 1H, H2´), 7.65-7.56 (m, 1H, H6´), 7.31 (t, 1H, *J* = 1.97 Hz, H5´), 7.18-7.11 (m, 1H, H4´); ^13^C-NMR δ: 160.7, 147.7, 144.7, 144.0, 142.4, 138.3, 134.8, 130.2, 124.9, 119.9, 117.7.

*6-Chloropyrazine-2-carboxylic acid (3-chlorophenyl)amide* (**2**). Yield: 91%; m.p. 107.0-108.0 °C; Anal. Calcd. for C_11_H_7_Cl_2_N_3_O (268.1): 49.28% C, 2.63% H, 15.67% N; found: 49.33% C, 2.61% H, 15.63% N; IR (cm^-1^): 3435 (N-H), 1676 (C=O); ^1^H-NMR δ: 9.44-9.35 (m, 2H, NH, H3), 8.82 (s, 1H, H5), 7.88 (t, 1H, *J* = 1.93 Hz, H2´), 7.60 (ddd, 1H, *J* = 7.97 Hz, *J* = 1.93 Hz, *J* = 0.83 Hz, H6´), 7.32 (t, 1H, *J* = 7.96 Hz, H5´), 7.17 (ddd, 1H, *J* = 7.97 Hz, *J* = 1.92 Hz, *J* = 0.82 Hz, H4´). ^13^C-NMR δ: 159.4, 147.8, 147.5, 143.6, 142.2, 137.9, 134.9, 130.2, 125.2, 120.1, 118.0.

*5-tert-Butylpyrazine-2-carboxylic acid (3-chlorophenyl)amide* (**3**). Yield: 83%; m.p. 117.0-118.0 °C; Anal. Calcd. for C_15_H_16_ClN_3_O (289.8): 62.18% C, 5.57% H, 14,50% N; found: 62.15% C, 5.51% H, 14.59% N; IR (cm^-1^): 3440 (N-H), 1685 (C=O);^1^H-NMR δ: 9.67 (bs, 1H, NH), 9.38 (d, 1H, *J* = 1.37 Hz, H3), 8.62 (d, 1H, *J* = 1.37 Hz, H6), 7.89 (t, 1H, *J* = 2.07 Hz, H2´), 7.59 (ddd, 1H, *J* = 7.96 Hz, *J* = 2.07 Hz, *J* = 1.10 Hz, H4´), 7.30 (t, 1H, *J* = 7.96 Hz, H5´), 7.13 (ddd, 1H, *J* = 7.96 Hz, *J* = 2.07 Hz, *J* = 1.10 Hz, H6´), 1.45 (s, 9H, CH_3_); ^13^C-NMR δ: 168.0, 161.1, 143.0, 141.0, 139.0, 138.5, 134.8, 130.1, 124.6, 120.45, 119.8, 117.6, 64.29, 37.1, 29.7.

*5-tert-Butyl-6-chloropyrazine-2-carboxylic acid (3-chlorophenyl)amide* (**4**). Yield: 97%; m.p. 86.0-87.0 °C; Anal. Calcd. for C_15_H_15_Cl_2_N_3_O (324.2): 55.57% C, 4.66% H, 12.96% N; found: 55.45% C, 4.63% H, 13.08% N; IR (KBr, cm^-1^): 3432 (N-H), 1678 (C=O); ^1^H-NMR δ: 9.39 (bs, 1H, NH), 9.26 (s, 1H, H3), 7.88 (t, 1H, *J* = 2.07 Hz, H2´), 7.60 (ddd, 1H, *J* = 7.97 Hz, *J* = 2.07 Hz, *J* = 1.10 Hz, H6´), 7.31 (t, 1H, *J* = 7.97 Hz, H5´), 7.15 (ddd, 1H, *J* = 7.97 Hz, *J* = 2.07 Hz, *J* = 1.10 Hz, H4´), 1.55 (s, 9H, CH_3_); ^13^C-NMR δ: 164.9, 159.9, 145.8, 140.7, 140.3, 138.2, 134.8, 130.1, 125.0, 120.0, 117.9, 116.79, 64.07, 39.0, 28.20.

*N-(2,6-dichlorophenyl)pyrazine-2-carboxamide* (**9**). Yield 66%; m.p. 151.0-152.0 °C; Anal. Calcd. for C_11_H_7_Cl_2_N_3_O (268.1): 49.28% C, 2.63% H, 15.67% N; found: 49.51% C, 2.68% H, 15.21% N; IR (cm^‑1^): 3377 (NH), 1685 (CO). ^1^H-NMR δ: 9.51 (bs, 1H, NH), 9.41 (s, 1H, H3), 8.85 (s, 1H, H5), 8.75 (s, 1H, H6), 7.10-7.61 (m, 3H, H3´, H4´, H5´); ^13^C-NMR δ: 160.8, 147.8, 144.8, 142.7, 134.2, 133.5, 132.4, 129.8, 128.8, 128.5, 123.5.

*6-Chloro-N-(2,6-dichlorophenyl)pyrazine-2-carboxamide* (**10**). Yield 78%; m.p. 178.0-179.2 °C; Anal. Calcd. for C_11_H_6_Cl_3_N_3_O (302.6): 43.67% C, 2.00% H, 13.89% N; found: 43.51% C, 1.98% H, 13.91% N; IR (cm^-1^): 3370 (NH), 1690 (CO); ^1^H-NMR δ: 9.41 (bs, 1H, NH), 9.38 (s, 1H, H3), 8.83 (s, 1H, H5), 7.12-7.52 (m, 3H, H3´, H4´, H5´); ^13^C-NMR δ: 159.3, 147.8, 147.4, 143.2, 142.1, 136.1, 132.9, 130.7, 130.6, 128.3, 121.5. 

*5-tert-Butyl-N-(2,6-dichlorophenyl)pyrazine-2-carboxamide* (**11**). Yield 43%; m.p. 53.5-55.0 °C. Anal. Calcd. for C_15_H_15_Cl_2_N_3_O (324.2): 55.57% C, 4.66% H, 12.69% N; found: 55.63% C, 4.71% H, 13.08% N; IR (cm^-1^): 3365 (NH), 1685 (CO); ^1^H-NMR δ: 9.67 (bs, 1H, NH), 9.37 (d, 1H, *J* = 1.37 Hz, H3), 8.61 (d, 1H, *J* = 1.37 Hz, H6), 7.12-7.48 (m, 3H, H3´, H4´, H5´), 1.45 (s, 9H, CH_3_); ^13^C-NMR δ: 168.2, 161.2, 143.2, 143.0, 142.1, 140.7, 139.0, 136.9, 133.0, 130.6, 127.7, 121.3, 118.9, 37.1, 29.7.

*5-tert-Butyl-6-chloro-N-(2,6-dichlorophenyl)pyrazine-2-carboxamide* (**12**). Yield 77%; m.p. 130.1-131.0 °C; Anal. Calcd. for C_15_H_14_Cl_3_N_3_O (358.7): 50.23% C, 3.93% H, 11.72% N; found: 50.33% C, 3.71% H, 12.08% N; IR (cm^-1^): 3390 (NH), 1685 (CO); ^1^H-NMR δ: 9.38 (bs, 1H, NH), 9.25 (s, 1H, H3), 7.12-7.48 (m, 3H, H3´, H4´, H5´), 1.55 (s, 9H, CH_3_); ^13^C-NMR δ: 165.1, 159.9, 145.8, 143.2, 142.1, 140.5, 140.3, 136.5, 133.0, 130.7, 128.2, 121.6, 119.1, 39.1, 28.2.

### 3.3. Lipophilicity determination by HPLC (capacity factor k/calculated log k)

Waters Alliance 2695 XE HPLC separation module and Waters Photodiode Array Detector 2996 (Waters Corp., Milford, MA, USA) were used. Waters Symmetry^®^ C_18_ 5 μm, 4.6 ° 250 mm, Part No. WAT054275 (Waters Corp., Milford, MA, USA) chromatographic column was used. The HPLC separation process was monitored by Empower™ 2 Chromatography Data Software, Waters 2009 (Waters Corp., Milford, MA, USA). The mixture of MeOH (HPLC grade, 70%) and H_2_O (HPLC–Mili-Q Grade, 30%) was used as a mobile phase. The total flow rate of the column was 1.0 mL/min, injection volume 30 μL, column temperature 30 °C and sample temperature 10 °C were used. The detection wavelength of 210 nm was chosen. The KI methanolic solution was used for the dead time (t_D_) determination. Retention times (t_R_) were measured in minutes.

The capacity factors *k* were calculated using the Empower™ 2 Chromatography Data Software according to formula *k* = (t_R_ - t_D_)/t_D_, where t_R_ is the retention time of the solute, whereas t_D_ denotes the dead time obtained using an unretained analyte. Log *k*, calculated from the capacity factor *k*, is used as the lipophilicity index converted to log *P* scale. The log *k* values of the individual compounds are shown in Table 1.

Distributive π parameters characterizing lipophilicity of the individual substituents were calculated according to the formula π = log *k*_S_ – log *k*_U_, where log *k*_S_ is the determined capacity factor logarithm of the individual substituted compounds, whereas log *k*_U_ denotes the determined capacity factor logarithm of the unsubstituted compound, it means π = 0. The determined pyrazine parameters π_Pyr_ of compounds **1**, **5**, **9** and **13** can be used as π_Pyr_ = 0. The determined π parameters of pyrazinecarboxamides with unsubstituted aniline (π_H_ = 0.4119, π_6-Cl_ = 0.6884, π*_t_*_-Bu_ = 0.9439, π_6-Cl+*t*-Bu_ = 1.2432) were used as π_Ph_ reference values. The distributive π parameters of the individual compounds are shown in Table 1.

### 3.4. Lipophilicity calculations

Log *P*, *i.e. ,* the logarithm of the partition coefficient for *n-*octanol/water, was calculated using the program ACD/LogP ver. 1.0 (Advanced Chemistry Development Inc., Toronto, Canada). The results are shown in Table 1.

### 3.5. In vitro antimycobacterial screening

Antimycobacterial evaluation was carried out in the tuberculosis antimicrobial acquisition and coordinating facility (TAACF), Southern Research Institute, Birmingham, AL, U.S.A., which is a part of the National Institutes of Health (NIH). Primary screening of all compounds was conducted at 6.25 µg/mL against *M. tuberculosis* H37Rv (ATCC27294) in BACTEC 12B medium using both BACTEC 460 radiometric system and the Microplate Alamar Blue Assay (MABA) [17,24]. For the results see Table 2.

### 3.6. In vitro antifungal susceptibility testing

The Department of Medical and Biological Sciences at the Faculty of Pharmacy in Hradec Králové, Charles University in Prague, Czech Republic, performed the antifungal susceptibility assays. The method used was the microdilution panel broth method with RPMI medium containing glutamine as a growth medium. Tested strains: *Candida albicans* ATCC 44859, *C . tropicalis* 156, *C . krusei* E28, *C. glabrata* 20/I, *Trichosporon asahii* 1188, *Trichophyton mentagrophytes* 445, *Aspergillus fumigatus* 231 and *Absidia corymbifera* 272. The values of the minimum inhibitory concentration (MICs) were determined after 24 and 48 h of static incubation at 35 °C and darkness [25]. For *T. mentagrophytes*, the final MICs were determined after 72 and 120 h of incubation. For the results of the most sensitive fungal strain *T. mentagrophytes*, see Table 2.

### 3.7. Study of inhibition photosynthetic electron transport (PET) in spinach chloroplasts

Chloroplasts were prepared from spinach (*Spinacia oleracea* L.) according to Masarovičová and Kráľová [26]. The inhibition of photosynthetic electron transport (PET) in spinach chloroplasts was determined spectrophotometrically (Genesys 6, Thermo Scientific, USA), using an artificial electron acceptor 2,6-dichlorophenol-indophenol (DCIPP) according to Kráľová *et al*. [27], and the rate of photosynthetic electron transport was monitored as a photoreduction of DCPIP. The measurements were carried out in phosphate buffer (0.02 mol/L, pH 7.2) containing sucrose (0.4 mol/L), MgCl_2_ (0.005 mol/L) and NaCl (0.015 mol/L). The chlorophyll content was 30 mg/L in these experiments and the samples were irradiated (~100 W/m^2^) from 10 cm distance with a halogen lamp (250 W) using a 4 cm water filter to prevent warming of the samples (suspension temperature 22 °C). The studied compounds were dissolved in DMSO due to their limited water solubility. The applied DMSO concentration (up to 4%) practically did not affect the photochemical activity in spinach chloroplasts (observed differences in DCPIP photoreduction due DMSO addition were within experimental error). The inhibitory efficiency of the studied compounds was manifested by IC_50_ values, *i.e.* by molar concentration of the compounds causing 50% decrease in the oxygen evolution rate relative to the untreated control. The comparable IC_50_ value for a selective herbicide 3-(3,4-dichlorophenyl)-1,1-dimethylurea, DCMU (Diurone^®^) was about 1.9 μmol/L [28]. The results are summarized in Table 2.

## 4. Conclusions

A series of sixteen ring-substituted *N*-phenylpyrazine-2-carboxamides were prepared by condensation of the corresponding chlorides of some substituted pyrazinecarboxylic acids (pyrazinecarboxylic acid, 6-chloropyrazine-2-carboxylic acid, 5-*tert*-butylpyrazine-2-carboxylic acid or 5-*tert*-butyl-6-chloropyrazine-2-carboxylic acid) with ring-substituted (chlorinated) anilines. The synthesis, analytical and spectroscopic data of newly prepared compounds are presented. Lipophilicity of the compounds was determined using a well characterized RP-HPLC method. The prepared compounds were tested for their ability to inhibit photosynthetic electron transport (PET) in spinach chloroplasts (*Spinacia oleracea* L.) and for their antifungal and antimycobacterial activity. 6-Chloro-*N*-(4-chlorophenyl)pyrazine-2-carboxamide (**6**) showed the highest activity against *M. tuberculosis* strain H37Rv (65% inhibition at 6.25 μg/mL). The highest antifungal effect (MIC = 62.5 μmol/L) against *Trichophyton mentagrophytes* was found for 6-chloro-5-*tert*-butyl-*N*-(3,4-dichlorophenyl)pyrazine-2-carboxamide (**16**). 6-Chloro-5-*tert*-butyl-*N*-(4-chlorophenyl) pyrazine-2-carboxamide (**8**) was the most active in the inhibition of photosynthetic electron transport (PET) in spinach (*Spinacia oleracea* L.) chloroplasts (IC_50_ = 43.0 μmol/L). The relationships between the lipophilicity and the chemical structure of the studied compounds as well as structure–activity relationships between the chemical structures and the antimycobacterial, antifungal and photosynthesis-inhibiting activities of the evaluated compounds are briefly discussed. The results of *in vitro* antimycobacterial and antifungal screening indicated the significance of lipophilicity of compounds. Correlations between PET-inhibiting activity and lipophilicity characteristics (log *k*, log *P*, π) of the compounds and Hammett's constants (σ) of the substituents on phenyl ring were performed. For the PET-inhibiting activity, the importance of compound lipophilicity was more significant than the electronic properties of the substituents expressed by σ values. Predominantly, the lipophilicity of substituents on the pyrazine was determinant.
